# Quantification of the Metabolic State in Cell-Model of Parkinson’s Disease by Fluorescence Lifetime Imaging Microscopy

**DOI:** 10.1038/srep19145

**Published:** 2016-01-13

**Authors:** Sandeep Chakraborty, Fang-Shin Nian, Jin-Wu Tsai, Artashes Karmenyan, Arthur Chiou

**Affiliations:** 1 Institute of Biophotonics, National Yang-Ming University, Taipei, Taiwan 11221, ROC; 2 Program in Molecular Medicine, National Yang-Ming University and Academia Sinica, Taipei, Taiwan 11529, ROC; 3 Institue of Brain Science, National Yang-Ming University, Taipei, Taiwan 11221, ROC; 4 Biophotonics and Molecular Imaging Research Center, National Yang-Ming University, Taipei, Taiwan 11221, ROC; 5 Department of Physics, National Dong Hwa University, Hualien, Taiwan 97401, ROC

## Abstract

Intracellular endogenous fluorescent co-enzymes, reduced nicotinamide adenine dinucleotide (NADH) and flavin adenine dinucleotide (FAD), play a pivotal role in cellular metabolism; quantitative assessment of their presence in living cells can be exploited to monitor cellular energetics in Parkinson’s disease (PD), a neurodegenerative disorder. Here, we applied two-photon fluorescence lifetime imaging microscopy (2P-FLIM) to noninvasively measure the fluorescence lifetime components of NADH and FAD, and their relative contributions in MPP^+^ (1-methyl-4-phenylpyridinium) treated neuronal cells, derived from PC12 cells treated with nerve growth factor (NGF), to mimic PD conditions. A systematic FLIM data analysis showed a statistically significant (p < 0.001) decrease in the fluorescence lifetime of both free and protein-bound NADH, as well as free and protein-bound FAD in MPP^+^ treated cells. On the relative contributions of the free and protein-bound NADH and FAD to the life time, however, both the free NADH contribution and the corresponding protein-bound FAD contribution increase significantly (p < 0.001) in MPP^+^ treated cells, compared to control cells. These results, which indicate a shift in energy production in the MPP^+^ treated cells from oxidative phosphorylation towards anaerobic glycolysis, can potentially be used as cellular metabolic metrics to assess the condition of PD at the cellular level.

Parkinson’s disease (PD) is the second most common neurodegenerative disease, next to Alzheimer’s disease, affecting at least one percent of population over the age of 70^1^. It is the most common movement disorder with cardinal signs of tremor, rigidity, akinesia, and problems with balance in humans^2^. PD is pathologically characterized by the progressive and extensive loss of dopaminergic neurons in substantia nigra pars compacta (SNpc), causing deficit of the neurotransmitter dopamine in the brain, which is essential for coordinated and controlled body movements^3^,^4^. Although it has been generally accepted that PD is a multifactorial disease, several studies have converged to mitochondrial dysfunction, which leads to the depletion of energy productions (adenosine 5′-triphosphate, ATP) in neurons due to inhibition of oxidative phosphorylation, as one of the fundamental causes of Parkinsonism^5^. In PD patients, complex I of the mitochondrial electron transport chain has been found to be defective, impairing the overall cellular respiration process, and eventually altering the cellular metabolism in affected neurons^6^,^7^.

The biochemical and molecular mechanisms involved in the dopaminergic neuronal cell death in PD remain elusive. Several neurotoxin based models have been developed over the last one and half decades to induce Parkinsonism in animal and cell models to elucidate the cause of PD^8^,^9^. Among them, the neurotoxin, 1-methyl-4-phenyl-1,2,3,6-tetrahydropyridine (MPTP)^10^, has been widely used. MPTP is in itself not cytotoxic; however, the enzyme monoamine oxidase B (MOAB) metabolizes MPTP to 1-methyl-4-phenylpyridinium (MPP^+^) after it crosses the blood-brain barrier^10^. Selective uptake of MPP^+^ by the dopaminergic neurons and further accumulation in mitochondria of the neurons in SNpc, causes damage to the complex I of the electron transport chain, and thereby affecting the cellular respiration^10^,^11^.

In cellular respiratory process, the autofluorescent intracellular coenzymes, reduced nicotinamide adenine dinucleotide (NADH) and flavin adenine dinucleotide (FAD), are the principal electron donor and acceptor in the electron transport chain^12^,^13^. Complex I serves as the substrate for NADH and complex II for FAD^14^,^15^. The ATP production in the mitochondria is accomplished through the transport of electrons to molecular oxygen through several complex enzymes- I, II, III, IV in the transport chain by the oxidation of NADH (to NAD^+^) and of the reduced flavin adenine dinucleotide (FADH_2_) to FAD, in complexes I and II, respectively^16^.

Although NADH and FAD exist both in oxidized and reduced forms in the cell, only the reduced NAD^+^ (i.e. NADH) and oxidized FADH_2_ (i.e. FAD) are fluorescent^17^. In 1962, Chance *et al.* demonstrated the possibility of utilizing the fluorescence properties of NADH to interpret the cellular metabolic state in terms of the relative amount of oxidized and reduced form of NADH^18^. Following this pioneering work, several studies to monitor cellular metabolic state in different diseases have been reported^19^,^20^. However, the application of this rather promising technique has remained relatively unexplored in the understanding of neurodegenerative diseases.

Till date, considerable advancement has been achieved in quantifying the autofluorescence of NADH and FAD through optical spectral and time-resolved methods. These methods have more advantages compared to chemical methods, wherein the cells are lysed to obtain the concentrations of redox couples such as pyruvates and lactates^21^. Thus, optical methods provide a way to study the cellular metabolic state in live cell and animal models in real time noninvasively, and thereby enabling the assessment of the levels of oxidative phosphorylation and glycolysis^22^. In fluorescence spectral based methods, fluorescence intensity can be measured to quantify the NADH/NAD^+^ and FADH_2_/FAD ratios^23^,^24^; such an approach, however, may contain artifacts due to the inhomogeneous concentrations of NADH and FAD in cells, and also to different quantum yields depending on free or protein-bound state of NADH (and FAD). All these shortcomings can be overcome by using the time-resolved fluorescence measurement techniques such as fluorescence lifetime imaging microscopy (FLIM)^25^,^26^.

Fluorescence lifetime can be defined as the average time the fluorophore remains in the excited state before descending to the ground state^27^,^28^. Fluorescence lifetime is an inherent and intrinsic property of a fluorophore, which is independent of the concentration of the fluorophore, as well as the quantum yield^26^. Besides, it is also rather insensitive to the excitation source intensity fluctuation, absorption and scattering. However, fluorescence lifetime is highly environment sensitive^29^. Thus, it can provide, in different cellular microenvironments, a contrast which is otherwise impossible to monitor by spectral measurements. This property of fluorescence lifetime was exploited in this study to differentiate and quantify the amounts of free and protein-bound NADH and FAD. The free NADH and protein-bound FAD have shorter lifetimes while the protein-bound NADH and free FAD have longer lifetimes^30^,^31^. The dynamic quenching of the nicotinamide moiety in NADH and flavin moiety in FAD by the adenine moiety yields shorter fluorescence lifetime^27^,^32^.

In this study, two-photon fluorescence lifetime imaging microscopy (2P-FLIM) was applied for functional mapping of the cellular redox state (NADH/NAD^+^ and FADH_2_/FAD ratios) in MPP^+^ treated neuronal cells, differentiated from PC12 cells, which were derived from the pheochromocytoma of the rat adrenal; this protocol serves as a model system of PD in laboratory conditions. PC12 cells have been widely used as a model system for neuronal based studies^33^,^34^ including Parkinson’s disease^35^, continuous NGF stimulation induces differentiation of these cells into neuron-like phenotypes^36^. The cellular redox state can be quantified in terms of the fluorescence lifetime components values of NADH and FAD, and also the relative contributions of free to protein-bound NADH (and FAD). Two-photon excitation was used for lifetime imaging of NADH and FAD, as it causes less photobleaching and yields higher cell viability^37^. Our results indicate a shift in cellular respiration towards anaerobic glycolysis in PD like conditions, resembling hypoxic conditions. This study and the results may serve as a precursor for further elucidating the cellular metabolism in tissue and *in vivo* conditions of PD and eventually as a distinct biomarker for discriminating the diseased cells in PD from the normal cells.

## Results

**Figure 1:**
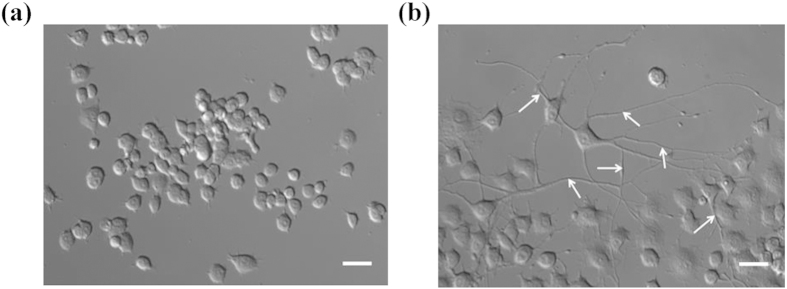
PC12 cells’ response to NGF (nerve growth factor) treatment. Differential interference contrast (DIC) microscopy images of (**a**) undifferentiated, and (**b**) differentiated PC12 cells after 200 ng/ml of NGF treatment for three days. The arrows in micrograph (**b**) point to the neuronal-like branching extended from PC12 cells after NGF treatment. Objective: 40x, NA 0.6, air. Scale bar: 20 μm.

In this study, as mentioned in the “Materials and methods” section, PC12 cells were treated with NGF; a change in cellular morphology from round and/or polygonal shapes in small clusters, before NGF treatment [Fig. 1(a)], into neuron-like phenotypes, with branching structures, after NGF treatment [Fig. 1(b)] was clearly observed. These differentiated PC12 cells were treated with MPP^+^, which is the active metabolite of MPTP (known to induce PD like conditions in cells as well as in animal models), to establish PD cellular model in this work.

In our experiments, optimization of the image acquisition parameters such as laser power and image acquisition time was established via photobleaching experiments. The results of the photobleaching experiments can be found in the Supplementary Figure S1.

**Figure 2:**
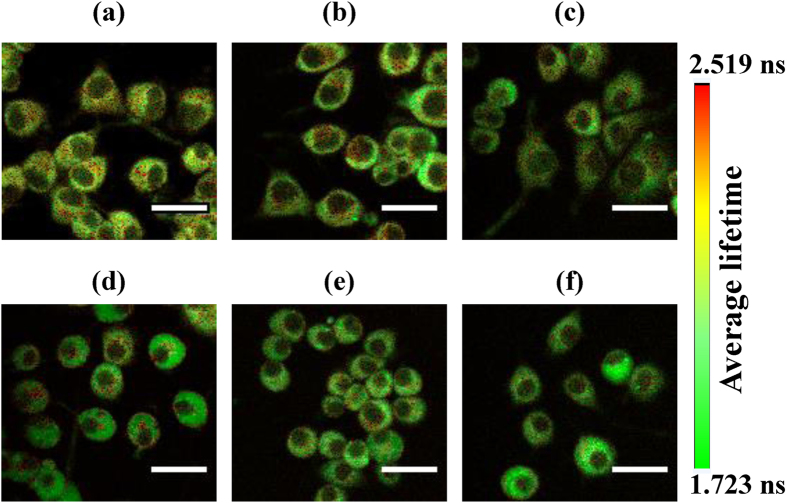
Micrographs of average NADH fluorescence lifetime. Pseudocolor mapping of average fluorescence lifetime (*τ*_*avg*_) of NADH of (**a**) the untreated differentiated PC12 cells, and of the cells treated with (**b**) 50, (**c**) 100, (**d**) 250, (**e**) 500, and (**f**) 1000 μM of MPP^+^. The color bar on the right represents the range of *τ*_*avg*_. Scale bar: 20 μm.

Representative average NADH fluorescence lifetime images of differentiated PC12 cells are shown in Fig. 2 for different concentrations of MPP^+^ treatment. The average lifetime was calculated using equation (2) in “Materials and methods” section. These images were taken with 760 nm excitation; and pseudocolor mapping over same range was utilized to represent the average lifetime distribution. From Fig. 2(a–f), it can be observed that the MPP^+^ treated cells show lower average lifetime as compared to the control cells; and the difference is clearly discernable, especially for the control cells [Fig. 2(a)] vs. the 1000 μM MPP^+^ treated cells [Fig. 2(f)].

**Figure 3:**
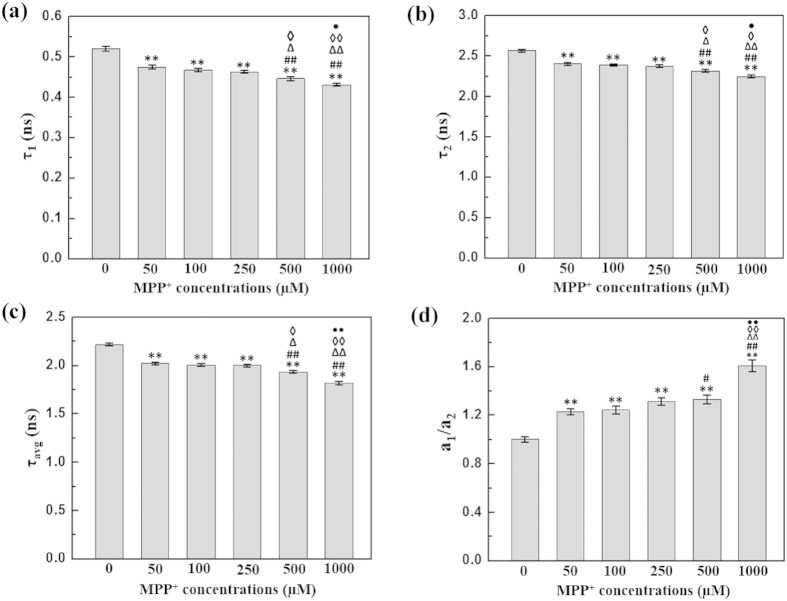
Summary of the effect of MPP^+^ on NADH fluorescence lifetime components. Average of all the data points (n = 75) from five experimental days of NADH lifetime measurements: (**a**) short, or free (*τ*_1_), (**b**) long, or protein-bound (*τ*_2_), (**c**) average (*τ*_*avg*_) NADH lifetimes, and (**d**) the ratio of the relative contribution of free, and protein-bound NADH (*a*_1_/*a*_2_) for cells treated with different concentrations of MPP^+^. The error bars indicate standard error of the mean (SEM). A one-way ANOVA with LSD-post-hoc analysis was performed to define statistical significance. Statistical significance: **p < 0.001, for control (0 μM MPP^+^) vs. MPP^+^ treated cells; #p < 0.05, ##p < 0.001, for 50 μM MPP^+^ treatment vs. other MPP^+^ treated cells; ^Δ^p < 0.05, ^ΔΔ^p < 0.001, for 100 μM MPP^+^ treatment vs. other MPP^+^ treated cells; ^◊^p < 0.05, ^◊◊^p < 0.001, for 250 μM MPP^+^ treatment vs. other MPP^+^ treated cells; ^●^p < 0.05, ^●●^p < 0.001, for 500 vs. 1000 μM MPP^+^ treatment.

All the experiments were performed independently for five experimental days, acquiring 15 data points per day. NADH fluorescence lifetime components, including the short (*τ*_1_), the long (*τ*_2_), and the average (*τ*_*avg*_), as well as the ratio of their contributions (*a*_1_/*a*_2_) are tabulated in Supplementary Table S1. Figure 3 shows the averaged values of all the data (75 data points) for each parameter. From control to 1000 μM MPP^+^ treated cells, *τ*_*avg*_ decreased by ~17% [Fig. 3(c)], while *a*_1_/*a*_2_ increased by 60% [Fig. 3(d)]; *τ*_1_ and *τ*_2_ also decreased by ~17 [Fig. 3(a)] and 11% [Fig. 3(b)], respectively. One-way analysis of variance (ANOVA) with least square difference (LSD) post-hoc analysis showed statistically significant differences among these values [Fig. 3].

**Figure 4:**
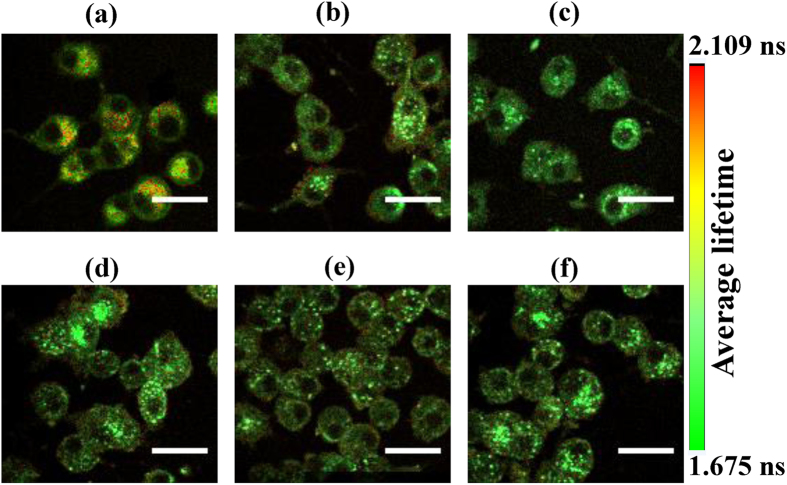
Average FAD fluorescence lifetime micrographs. Pseudo-color mapping of FAD average fluorescence lifetime (*τ*_*avg*_) at (**a**) 0, (**b**) 50, (**c**) 100, (**d**) 250, (**e**) 500, and (**f**) 1000 μM MPP^+^ treatment of differentiated PC12 cells. The color bar shows the range of the FAD average lifetime. Scale bar: 20 μm.

Figure 4 shows the representative average fluorescence lifetime images of FAD at different concentrations of MPP^+^ treatment of the differentiated PC12 cells. FAD fluorescence was excited at 860 nm and pseudo-color mapping of average life time was used to show its distribution. From Fig. 4(a–f), one can infer that the average lifetime of FAD also decreased from the control cells to the MPP^+^ treated cells. However, the differences in FAD lifetime distribution among the PD induced cells were much smaller compared with the corresponding results for NADH.

**Figure 5:**
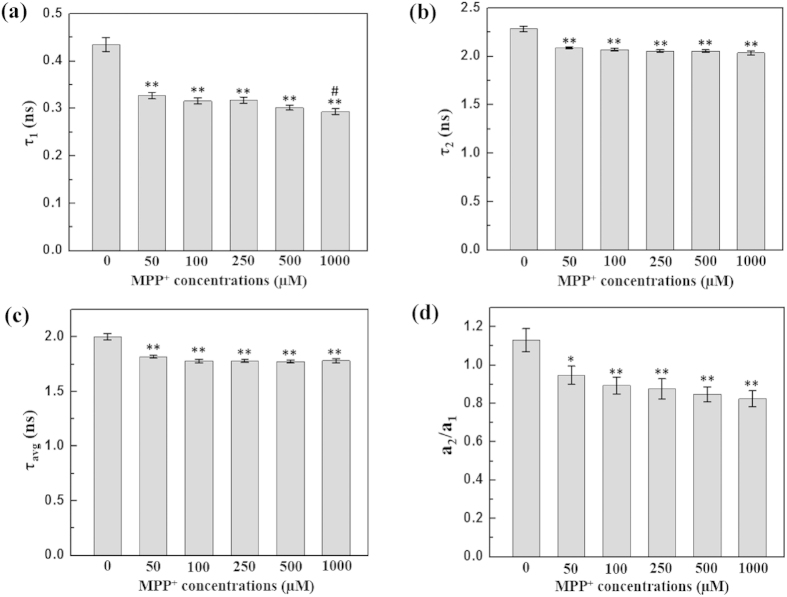
Summary of the effect of MPP^+^ on FAD fluorescence lifetime components. Average and SEMs (error bars) of all the 75 data points from all the imaging sessions of five experimental days of fluorescence lifetime mapping: (**a**) short, or protein-bound (*τ*_1_), (**b**) long, or free (*τ*_2_), (**c**) average (*τ*_*avg*_) FAD lifetimes, and (**d**) the ratio of the relative contribution of free, and protein-bound FAD (*a*_2_/*a*_1_) as a function of MPP^+^ concentration. A one-way ANOVA with LSD post-hoc analysis based statistical significance: *p < 0.05, **p < 0.001, for control (0 μM MPP^+^) vs. MPP^+^ treated cells; #p < 0.05 for 50 μM MPP^+^ treatment vs. Other MPP^+^ treated cells.

The averaged values of all data points for each parameter of lifetime components analysis of FAD are shown in Fig. 5. The values for each experimental days can be found in Supplementary Table S2. The short (protein-bound), long (free), and average fluorescence lifetimes of FAD, i.e., (*τ*_1_), (*τ*_2_), and (*τ*_*avg*_), decreased by ~32% [Fig. 5(a)], ~10% [Fig. 5(b)], and 11% [Fig. 5(c)], respectively, from control to 1000 μM MPP^+^ treated cells. The relative contributions of free- to protein-bound FAD (*a*_2_/*a*_1_) also decreased by ~27% [Fig. 5(d)], indicating a rise in the protein-bound FAD component. However, among the MPP^+^ treated cells, the differences in their free [Fig. 5(a)], protein-bound [Fig. 5(b)], average FAD lifetimes [Fig. 5(c)] and a_2_/a_1_ [Fig. 5(d)] for FAD were statistically insignificant.

## Discussion

In this study, functional mapping of intracellular reduction-oxidation states in PD cellular model was accomplished using 2P-FLIM of autofluorescent co-enzymes NADH and FAD. Recently, Plotegher *et al.*^38^ have reported the effect of alpha synuclein aggregation *in vitro* and in human embryonic kidney 293 cell line (which is not a neuronal cell line) using FLIM technique. However, to investigate PD, it would be more proper to study the effect in dopaminergic neurons or other neuron-like cells. Hence, to the best of our knowledge, ours is the first experimental study to apply FLIM technique to PD cell model to elucidate the reduction-oxidation state in neurodegenerative diseases at the cellular level. Our experimental results show that the average as well as the free and protein-bound fluorescence lifetimes of both NADH and FAD decreased significantly in MPP^+^ treated cells. On the other hand, in the PD induced cells, the relative contribution of free to protein-bound NADH increased while the same ratio decreased for FAD. As NADH and FAD are the major electron carriers in the energy production of cells, these results can be used to characterize quantitatively the time-development of the cellular redox state^17^. In this study, the major reduction-oxidation ratios (redox ratios), NADH/NAD^+^ and FADH_2_/FAD, in neuronal cells, derived from PC12 cells, with vs. without MPP^+^ treatment were evaluated in terms of the ratios of free-to-protein-bound NADH (*a*_1_/*a*_2_) and FAD (*a*_2_/*a*_1_). The change in these ratios, which directly reflects the change in cellular metabolic state, may serve as important biomarkers in diagnostic and/or therapeutic applications in PD.

It is of utmost important to develop PD models to mimic its pathological conditions in laboratory environment to study its etiology, and for potential therapeutic applications. MPTP is a PD neurotoxin which has been used to induce PD syndromes in cells and animal models and mostly used to understand the mechanisms involved in PD. In our study, we have treated the neuronal cells, obtained from PC12 cells, with MPP^+^ to mimic the PD conditions and quantified its metabolic state through noninvasive FLIM technique. Our results show that *a*_1_/*a*_2_ increased (for NADH) while *a*_2_/*a*_1_ decreased (for FAD) significantly from the control to the 1000 μM MPP^+^ treated neuronal cells, derived from PC12 cells (p < 0.001). These results indicate a rise in the relative contributions of free NADH and protein-bound FAD components in PD induced cells. Previous studies have shown that MPP^+^ is a Complex I inhibitor of the electron transport chain, which inhibits the transfer of electrons from electron donors to NADH; as a result, oxidation of NADH is impaired, and the level of free NADH pool is raised subsequently^10^. Moreover, MPP^+^ may also partially inhibit the activities of Complex II (succinate dehydrogenase; SDH), which serves as the substrate for FAD^39^. Complex II is the only enzyme in the inner mitochondrial membrane which takes part in both citric acid cycle (Krebs cycle) and electron transport chain. It is the second entry point of electrons in the electron transport chain via FADH_2_. Through the oxidation of succinate (which directly binds to the subunit SDHA of complex II) to fumarate, electrons are transported to FAD, incorporated in SDHA, thus reducing it to FADH_2_^40^,^41^. Our results also show an increase in protein-bound FAD component which, in this case, can be due to the inhibition of SDHA or SDHB subunit which prohibits the reduction of protein-bound FAD to FADH_2_^42^. Thus, an increase in *a*_1_/*a*_2_ (in NADH) along with a decrease in *a*_2_/*a*_1_ (in FAD), jointly reflect a lower metabolic activity in the PD induced cells. Overall, all these previous results are consistent with our results and interpretations.

MPP^+^, like rotenone and potassium cyanide (KCN), is an inhibitor of electron transport chain. Bird *et al.*^43^ and Schneckenburger *et al.*^44^ showed that the average fluorescence lifetime of NADH decreased, in MCF10A human breast cells and in BKeZ-7 endothelial cells, upon treatment of KCN and rotenone, respectively. MPP^+^ also exhibited similar effect in our PC12 cells based PD model wherein the free and protein-bound NADH fluorescence lifetime as well as the average fluorescence lifetime decreased significantly from control to 1000 μM MPP^+^ treated cells (P < 0.001) [Fig. 4(c)]. Previous literature also showed that a decrease in fluorescence lifetime, and an increase in the relative contribution of free vs. protein-bound NADH may indicate hypoxic condition in cells^44^. Rotenone and KCN inhibit the transfer of electrons from NADH to oxygen, whereas hypoxic conditions lower the level of oxygen that can accept the electrons. We can thus speculate that the decrease in fluorescence lifetime of NADH can probably be related to the increase in the NADH/NAD^+^ ratio in MPP^+^ treated cells as well. Moreover, the protein-bound NADH fluorescence lifetime can either increase or decrease depending on its binding sites^45^,^46^. In our study, the decrease in the protein-bound NADH fluorescence lifetime can also be attributed to the change in its binding site in complex I^47^, where dynamic quenching of the nicotinamide moiety by adenine moiety might be the cause of free NADH lifetime decrease in the MPP^+^ treated cells, indicating that the cells were in hypoxic conditions^32^,^43^,^44^. These mechanisms also reflect the possibility of a shift in cellular ATP production from oxidative phosphorylation to anaerobic glycolysis. Hence, it can be stated that in our cellular PD model the energy production is principally regulated through anaerobic glycolysis. This conclusion is consistent with earlier works wherein it was clearly demonstrated that the inhibition of complex I of electron transport chain by MPP^+^ might lead to ATP production through anaerobic glycolysis^48^,^49^.

To further validate our conclusion (from the lifetime data analysis) of shifting cellular respiration from oxidative phosphorylation towards anaerobic glycolysis in our PD cellular model, oxygen consumption rate (OCR) assay together with extracellular acidification rate (ECAR) assay were performed following the protocol from Seahorse biosciences (Massachusetts, USA). Oxygen consumption rate is an indicator of mitochondrial respiration, while the extracellular acidification rate indicates the lactic acid formation during glycolytic energy metabolism. In our MPP^+^ treated cells, the OCR decreased significantly (P < 0.001) from the control cells, while ECAR increased significantly (P < 0.001) (Supplementary Fig. S4). These results further substantiate the shift of cellular respiration from oxidative phosphorylation towards anaerobic glycolysis in MPP^+^ treated cells. Hence, the decrease in *a*_1_/*a*_2_ (in NADH) and increase in *a*_2_/*a*_1_ (in FAD) ratios can indicate the preference of cellular respiration towards anaerobic glycolysis, than oxidative phosphorylation in MPP^+^ treated cells, and in hypoxia.

FAD is one of the most important cofactors of flavins. In our PD model, the range of the free, and protein-bound FAD fluorescence lifetimes along with their average value are consistent with the results (in solutions^31^ and in eye^50^) published in the literatures. The effect of mitochondrial electron chain inhibitors on FAD lifetime has not yet been reported in the literatures. In our PC12 cell based PD model, the protein-bound FAD lifetime decreased while their relative contribution increased [Fig. 5]. NAD^+^ level directly affects the protein-bound FAD lifetime, bearing a negative correlation to each other^32^. Thus, a decrease in the protein-bound FAD lifetime indicates a higher level of NAD^+^ in PD induced cells relative to the control cells, although the relative contribution of free NADH is much higher compared to NAD^+^ level as is indicated by the ratio (*a*_1_/*a*_2_), i.e., free/protein-bound NADH.

MPP^+^ impairs the oxidative phosphorylation in a dose- and time- dependent manner^11^. In this work, we also investigated the dose-dependent effect of MPP^+^ on the fluorescence lifetime of NADH and FAD. Previous studies showed a concentration-dependent effect of MPP^+^ on cellular functionality for concentrations ranging from 10 μM through 1 mM^51^. In our study, the MPP^+^ concentration was varied from 50, 100, 250, 500, 1000 μM. Statistically, no significant difference was observed in free, protein-bound, and average NADH lifetimes among 50, 100, and 250 μM MPP^+^ treated cells [Fig. 3(a–c)]. Likewise, no significant difference was observed in the relative contributions of free to protein-bound NADH among 50, 100 and 250 μM MPP^+^ treated cells [Fig. 3(d)]. The reasons for this concentration-independence of NADH lifetime components remain elusive. However, it can be speculated that in this concentration range the NADH/NAD^+^ ratio was not affected significantly. Moreover, the relative contribution of FAD fluorescence lifetime as well as its average lifetime did not vary significantly with change in MPP^+^ concentrations. This can be attributed to the concentration-independence of NADH lifetimes as FAD is always in dynamic equilibrium with the mitochondrial NADH/NAD^+^ pool^51^.

In this study, the fluorescence lifetime analysis was done by taking into account all the pixels in the cytoplasm (including mitochondria) of a cell or, a group of cells; as the fluorescence signal of NADH in cytoplasm is much less compared to that in the mitochondria^52^,^53^ which is also evident in our case, (see Supplementary Fig. S5) and the primary location of FAD is in mitochondria, the contribution from the cytoplasm can be assumed to be negligible. Previous studies also demonstrated that the fluorescence lifetime calculated by averaging over a few pixels in cytoplasm did not differ significantly with the corresponding value obtained by averaging over all the pixels in the cytoplasm^43^,^54^. Thus, our approach of calculating the fluorescence lifetime is adequate to provide values which are reliable and consistent.

We have thus demonstrated experimentally the possibility of utilizing time-resolved two-photon fluorescence lifetime imaging to monitor the NADH and FAD autofluorescence lifetimes for potential identification and disease progression assessment of PD. This approach can be further extended to the study of PD in tissue model and *in vivo* in animal model, with the potential for early detection of PD without the necessity of any exogenous dyes.

## Conclusion

We report the measurement of NADH and FAD fluorescence lifetime components as well as their contribution in PC12 cells differentiated into neuronal phenotype, and subsequently treated with MPP^+^, to serve as a cell model system for PD studies. MPP^+^ induced reduction-oxidation changes correlate highly with the observed changes in *a*_1_/*a*_2_ (for NADH); and *a*_2_/*a*_1_ (for FAD) as well as their respective lifetimes. Since the main effects of MPP^+^, in selectively antagonizing the dopaminergic neurons through blocking of electron transport chain, not only in cellular model, but also in animal models as well in humans, our approach may pave a way to further shed light on the molecular mechanisms of PD pathogenesis. Successful mapping of intracellular redox state in PD cellular model through time-resolved technique can potentially serve as a forerunner for the assessment of different stages of PD progression *in vitro* as well as *in vivo* and also in developing new diagnostic tools.

## Materials and Methods

### Cell culture

PC 12 cells were grown in RPMI medium 1640 (Gibco, Life technologies, Massachusetts, USA) supplemented with 10% horse serum, 5% fetal bovine serum and cultured in a water-saturated atmosphere at 37 °C and 5% CO_2_. To induce differentiation, cells were plated onto 50 μg/ml laminin/0.1 mg/ml poly-l-lysine (Sigma-Aldrich, St. Louis, USA) coated glass bottom 35 mm cell culture dishes and treated with 200 ng/ml nerve growth factor-7S (NGF-7S, derived from murine submaxillary gland) (Sigma-Aldrich, St. Louis, USA) for three days. Following this, cells were treated with different concentrations (0, 50, 100, 250, 500, and 1000 μM) of MPP^+^ iodide (Sigma-Aldrich, St. Louis, USA). These cells, with and without MPP^+^ treatment, were used for NADH and FAD autofluorescence imaging by FLIM. The microscope stage is equipped with temperature control to maintain the cells at 37 °C; besides, cells were transferred to loading buffer (with 2.2 mM CaCl_2_.H_2_O, 150 mM NaCl, 5 mM KCl, MgCl_2_.H_2_O, HEPES 10 mM, 5 mM glucose, pH 7.4, Osm300) to maintain the physiological conditions during image acquisition.

### 2P-FLIM instrumentation

A mode-locked Ti:sapphire laser (Tsunami, Spectra-Physics, USA), capable of producing picosecond laser pulses with repetition rate of 82 MHz in the spectral range from 740 to 920 nm, pumped by a 5 W solid-state frequency-doubled 532 nm Millennia Vs (Spectra-Physics, USA) laser, was used as the two-photon fluorescence excitation source for the lifetime imaging of NADH and FAD (Supplementary Fig. S2). All the experiments were performed on an inverted Olympus microscope IX 71 (Olympus, Japan), equipped with a 2D galvanometer scanning unit (Einst Technology Pte Ltd, Singapore) for the two dimensional scanning of our samples. The laser beam was focused on the sample with a 60x, 1.20 numerical aperture (NA) plan apochromatic water immersion objective (UPlanSApo, Olympus, Japan). An average laser power of ~4 to 5 mW (after the objective) was used for fluorescence excitation to avoid photo-damage to the cells as well as photobleaching of NADH and FAD (Supplementary Fig. S1). Several studies have showed that the two-photon excitation wavelength range of 720–760 nm is good enough to selectively excite NADH, while the same for FAD is above 800 nm with a broad peak around 900 nm^51^,^55^,^56^. In this work, for two-photon excitation of NADH and FAD, the central wavelength of the laser was set at 760 and 860 nm respectively. All the emission signals were collected in a de-scanned mode of the microscope and detected by a photomultiplier tube (PicoQuant Gmbh, Berlin, Germany). Following the spectral characteristics of the autofluorescence molecules, band-pass filters, 447/60 and 525/50 nm from Semrock (New York, USA), were used for the detection of NADH and FAD fluorescence emission signals, respectively. A short-pass infrared cutoff filter was further used in the detection path to block the back-scattering of the excitation light. All the images were collected with 256 × 256 pixels resolution and each image was collected for ~800 seconds to accumulate sufficient number of photons for data analysis.

In our experimental set up, the fluorescence lifetime was measured through time-domain technique viz. time-correlated single photon counting (TCSPC). Here, the time difference between the excitation and corresponding emitted photon was recorded via high-speed electronics. The fluorescence emission signal and a reference signal (laser excitation pulse) from a photodiode (TDA 200, PicoQuant GmbH, Berlin, Germany) were coupled to the electronic board of TCSPC, PicoHarp 300 (PicoQuant GmbH, Berlin, Germany). The electronics functions in a reverse START-STOP mode to record the photon arrival times where the START signal was generated via the detector PMT by the emission photons, and the STOP signal was from TDA 200. To enable fully a time-resolved analysis of the NADH and FAD autofluorescence signals, the PicoHarp was always operated in time-tagged time-resolved (TTTR) mode.

### 2P-FLIM data analysis

The FLIM data obtained as described above was analyzed by a commercially available software package SymPho Time, Version 5.2.4.0 (PicoQuant GmbH, Berlin, Germany). A lifetime measurement, from a decay curve and thereby extracting physically meaningful informations, involves the curve-fitting of a decay model function to the experimental data. Here, the model function was first convolved with an experimental instrument response function (IRF), before it was curve-fitted with the experimental data. However, due to the presence of background noise from the dark counts of detector, and/or a temporal shift between IRF and decay (originated from the wavelength dependence of photon counting units), a non-linear least-square fitting (assuming Poisson noise of data) was used *in lieu of* direct fitting approach. The goodness of fit was accepted as long as the chi square value remains ~1 and the residues showed random pattern; which eventually paved the way for unbiased data analysis (Supplementary Fig. S3). In this study, the IRF was estimated from the second harmonic generation (SHG) signal of a thin urea film and was calculated to be 0.181 ns. The double exponential model function, used for fitting to quantify the lifetime components of NADH and FAD, is as follows,


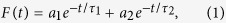


where, the parameters *τ*_1_ and *τ*_2_ represent the short and long lifetime components of NADH, while the coefficients *a*_1_ and *a*_2_ represent the fractional relative amplitudes, or contributions of these two different lifetime components, respectively. Similar interpretation was also assigned for FAD wherein *τ*_1_ and *τ*_2_ denoted the long and short lifetime components and the respective coefficients provided their amplitudes level. The ratios *a*_1_/*a*_2_ (for NADH) and *a*_2_/*a*_1_ (for FAD) were used to compare the relative contributions of the free and bound forms of these autofluorescent coenzymes^57^. The average fluorescence lifetime was defined as follows,


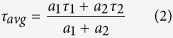


The double exponential model function was adequate to fit the lifetime decay curves of both NADH and FAD, as several studies have already showed the existence of two distinct lifetime components of these endogenous fluorophores^30^,^31^,^44^,^58^.

In this study, at least five images of cultured cells were acquired per 35 mm cell culture dish. Thus, the total number of images acquired per experimental day, including control (1 dish) and MPP^+^ treated cells (5 dishes) were 30 (i.e. 5 images x 6 dishes). The analysis of FLIM data included a region of interest (ROI) of the cytoplasm of one cell or several cells grouped together, excluding their nuclei. All the pixels in the cytoplasmic region were taken into account in the analysis. For proper fitting, more than 1000 counts of photons per pixel were ensured after binning, and the data were analyzed via pixel-by-pixel. Each set of experimental conditions was performed independently for five experimental days and the results were represented as mean ± standard error of mean (SEM).

## Statistical Analysis

A one-way analysis of variance (ANOVA) with least significant difference (LSD) post-hoc analysis was performed to evaluate variance and the significance of differences in fluorescence lifetime components as well as their relative contributions among the control and the MPP^+^ treated cells. The critical p-value for One-way ANOVA with LSD post-hoc analysis was set at 0.05.

## Additional Information

**How to cite this article**: Chakraborty, S. *et al.* Quantification of the Metabolic State in Cell-Model of Parkinson's Disease by Fluorescence Lifetime Imaging Microscopy. *Sci. Rep.* **6**, 19145; doi: 10.1038/srep19145 (2016).

## Supplementary Material

Supplementary Information

## References

[b1] Pringsheim T., Jette N., Frolkis A. & Steeves T. D. L. The prevalence of Parkinson’s disease: a systematic review and meta-analysis. Mov. Disord. 29(13), 1583–1590 (2014). 24976103 10.1002/mds.25945

[b2] Bereczki D. The description of all four cardinal signs of Parkinson’s disease in a Hungarian medical text published in 1690. Parkinsonism Relat. Disord. 16(4), 290–293 (2010). 19948422 10.1016/j.parkreldis.2009.11.006

[b3] Dauer W. & Przedborski S. Parkinson’s disease: mechanisms and models. Neuron 39, 889–909 (2003). 12971891 10.1016/s0896-6273(03)00568-3

[b4] Lang A. E. & Lozano A. M. Parkinson’s disease: first of two parts. N. Engl. J. Med. 339(15), 1044–1052 (1998). 9761807 10.1056/NEJM199810083391506

[b5] Winkhofer K. F. & Haass C. Mitochondrial dysfunction in Parkinson’s disease. Biochim. Biophys. Acta 1802, 29–44 (2010). 19733240 10.1016/j.bbadis.2009.08.013

[b6] Schapira A. H. V. Evidence for mitochondrial dysfunction in Parkinson’s disease- a critical appraisal. Mov. Disord. 9(2), 125–138 (1994). 8196673 10.1002/mds.870090202

[b7] Schultz J. B. & Beal M. F. Mitochondrial dysfunction in movement disorders. Curr. Opin. Neurol. 7(4), 333–339 (1994). 7952242 10.1097/00019052-199408000-00010

[b8] Bove J. & Perier C. Neurotoxin-based models of Parkinson’s disease. Neuroscience 211, 51–76 (2012). 22108613 10.1016/j.neuroscience.2011.10.057

[b9] Martinez T. N. & Greenamyre J. T. Toxin models of mitochondrial dysfunction in Parkinson’s disease. Antioxid. Redox Signal 16(9), 920–934 (2012). 21554057 10.1089/ars.2011.4033 PMC3292753

[b10] Przedborski S. & Vila M. MPTP: a review of its mechanisms and neurotoxicity. Clin. Neurosci. Res. 1, 407–418 (2001).

[b11] Przedborski S., Tieu K., Perier C. & Vila M. J. Bioenerg. MPTP as a mitochondrial neurotoxic model of Parkinson’s disease. Biomembr. 36(4), 375–379 (2004). 10.1023/B:JOBB.0000041771.66775.d5 15377875

[b12] Lehninger A. L. Phosphorylation coupled to oxidation of dihydrodiphosphopyridine nucleotide. J. Biol. Chem. 190, 345–359 (1951). 14841183

[b13] Kim H. J. & Winge D. R. Emerging concepts in the flavinylation of succinate dehydrogenase. Biochim. Biophys. Acta. 1827(5), 627–636 (2013). 23380393 10.1016/j.bbabio.2013.01.012 PMC3626088

[b14] Sazanov L. A. A giant molecular proton pump: structure and mechanism of respiratory complex I. Nat. Rev. Mol. Cell Biol. 16, 375–388 (2015). 25991374 10.1038/nrm3997

[b15] Kearny E. B. Studies on Succinic dehydrogenase. J. Biol. Chem. 235(3), 865–877 (1960). 14405058

[b16] Chance B. & Williams G. R. The respiratory chain and oxidative phosphorylation. Adv. Enzymol. Relat. Subj. Biochem. 17, 65–134 (1956). 10.1002/9780470122624.ch2 13313307

[b17] Heikal A. A. Intracellular coenzymes as natural biomarkers for metabolic activities and mitochondrial anomalies. Biomark. Med. 4(2), 241–263 (2010). 20406068 10.2217/bmm.10.1 PMC2905054

[b18] Chance B., Cohen P., Jobsis F. & Schoener B. Intracellular oxidation-reduction state *in vivo*. Science 137, 499–508 (1962). 13878016 10.1126/science.137.3529.499

[b19] Skala M. C. *et al.* *In vivo* multiphoton microscopy of NADH and FAD redox states, fluorescence lifetimes, and cellular morphology in precancerous epithelia. Proc. Natl. Acad. Sci. USA 104(49), 19494–19499 (2007). 18042710 10.1073/pnas.0708425104 PMC2148317

[b20] Quinn K. P. *et al.* Quantitative metabolic imaging using endogenous fluorescence to detect stem cell differentiation. Sci. Rep. 3, 3432 (2013). 24305550 10.1038/srep03432 PMC3851884

[b21] Williamson D. H., Lund P. & Krebs H. A. The redox state of free nicotinamide-adenine dinucleotide in the cytoplasm and mitochondria of rat liver. Biochem. J. 103(2), 514–527 (1967). 4291787 10.1042/bj1030514 PMC1270436

[b22] Skala M. & Ramanujam N. Multiphoton redox ratio imaging for metabolic monitoring *in vivo*. Methods Mol. Biol. 594, 155–162 (2010). 20072916 10.1007/978-1-60761-411-1_11 PMC2874879

[b23] Kasischke K. A., Vishwasrao H. D., Fisher P. J., Zipfel W. R. & Webb W. W. Neural activity triggers neuronal oxidative metabolism followed by astrocytic glycolysis. Science 305(5680), 99–103 (2004). 15232110 10.1126/science.1096485

[b24] Ostrander J. H. Optical redox ratio differentiates breast cancer cell lines based on estrogen receptor status. Cancer Res. 70(11), 4759–4766 (2010). 20460512 10.1158/0008-5472.CAN-09-2572 PMC3826951

[b25] Becker W. Fluorescence lifetime imaging- techniques and applications. J. Microsc. 247(2), 119–136 (2012). 22621335 10.1111/j.1365-2818.2012.03618.x

[b26] Berezin Y. & Achilefu S. Fluorescence lifetime measurements and biological imaging. Chem. Rev. 110, 2641–2648 (2010). 20356094 10.1021/cr900343z PMC2924670

[b27] McGown L. B. & Nithipatikom K. Molecular fluorescence and phosphorescence. Appl. Spectrosc. Rev. 35(4), 353–393 (2000).

[b28] Jablonski A. Über den Mechanisms des Photolumineszenz von Farbstoffphosphoren. Z. Phys. 94, 38–46 (1935).

[b29] Chen Y. E. & Periasamy A. Characterization of two-photon excitation fluorescence lifetime imaging microscopy for protein localization. Microscopy Res. Tech. 63, 72–80 (2004). 10.1002/jemt.10430 14677136

[b30] Lakowicz J. R., Szmacinski H., Nowaczyk K. & Johnson M. L. Fluorescence lifetime imaging of free and protein-bound NADH. Proc. Natl. Acad. Sci. USA 89, 1271–1275 (1992). 1741380 10.1073/pnas.89.4.1271 PMC48431

[b31] Nakashima N., Yoshihara K., Tanaka F. & Yagi K. Picosecond fluorescence lifetime of the coenzyme of D-amino acid oxidase. J. Biol. Chem. 255(11), 5261–5263 (1980). 6102996

[b32] Maeda-Yorita K. & Aki K. Effect of nicotinamide adenine dinucleotide on the oxidation-reduction potentials of lipoamide dehydrogenase from pig heart. J. Biochem. (Tokyo) 96, 683–690 (1984). 6548741 10.1093/oxfordjournals.jbchem.a134886

[b33] Westerink R. H. S. & Ewing A. G. The PC12 cell as model for neurosecretion. Acta Physiol. (Oxf.) 192(2), 273–285 (2008). 18005394 10.1111/j.1748-1716.2007.01805.x PMC2663028

[b34] Zhou T., Xu B., Que H., Lv S. & Liu S. Neurons derived from PC12 cells have the potential to develop synapses with primary neurons from rat cortex. Acta Neurobiol. Exp. 66, 105–112 (2006). 10.55782/ane-2006-1596 16886720

[b35] Grau C. M. & Greene L. A. Use of PC12 cells and rat superior cervical ganglion sympathetic neurons as models for neuroprotective assays relevant to Parkinson’s disease. Methods Mol. Biol. 846, 201–211 (2012). 22367813 10.1007/978-1-61779-536-7_18 PMC3678375

[b36] Greene L. A. & Tischler A. S. Establishment of noradrenergic clonal line of rat adrenal pheochromocytoma cells which respond to nerve growth factor. Proc. Natl. Acad. Sci. USA 73(7), 2424–2428 (1976). 1065897 10.1073/pnas.73.7.2424 PMC430592

[b37] Benninger R. K. P. & Piston D. W. Two-photon excitation microscopy of the study of living cells and tissues. Curr. Protoc. Cell Biol. 59, 4 Unit:4.1124 (2013). 10.1002/0471143030.cb0411s59 PMC4004770 23728746

[b38] Plotegher N. *et al.* NADH fluorescence lifetime is an endogenous reporter of α-synuclein aggregation in live cells. FASEB J. 29, 2484–2494 (2015). 25713058 10.1096/fj.14-260281 PMC4447231

[b39] Mizuno Y., Sone N. & Saitoh T. Effects of 1-methyl-4-phenyl-1,2,3,6-tetrahydropyridine and 1-methyl-4-phenylpyridinium ion on activities of the enzymes in the electron transport system in mouse brain. J. Neurochem. 48, 1787–1793 (1987). 3106573 10.1111/j.1471-4159.1987.tb05737.x

[b40] Rutter J., Winge D. R. & Schiffman J. D. Succinate dehydrogenase-assembly, regulation and role in human disease. Mitochondrion 10, 393–401 (2010). 20226277 10.1016/j.mito.2010.03.001 PMC2874626

[b41] Sun F. *et al.* Crystal structure of mitochondrial respiratory membrane protein complex II. Cell 121, 1043–1057 (2005). 15989954 10.1016/j.cell.2005.05.025

[b42] Grimm S. Respiratory chain complex II as general sensor for apoptosis. Biochim. Biophys. Acta-Bioenerg. 1827, 565–572 (2013). 10.1016/j.bbabio.2012.09.009 23000077

[b43] Bird D. K. *et al.* Metabolic mapping of MCF10A breast cells via multiphoton fluorescence lifetime imaging of the coenzyme NADH. Cancer Res. 65(19), 8766–8733 (2005). 16204046 10.1158/0008-5472.CAN-04-3922

[b44] Schneckenburger H., Wagner M., Weber P., Strauss W. S. L. & Sailer R. Autofluorescence lifetime imaging of cultivated cells using a UV picosecond laser diode. J. Fluorescence 14(5), 649–654 (2004). 10.1023/b:jofl.0000039351.09916.cc 15617271

[b45] Gafni A. & Brand L. Fluorescence decay studies of reduced nicotinamide adenine dinucleotide in solution and bound to liver alcohol dehydrogenase. Biochemistry 15(15), 3165–3171 (1976). 8086 10.1021/bi00660a001

[b46] Jameson D. M., Thomas V. & Zhou D. M. Time-resolved fluorescence studies on NADH bound to mitochondrial malate dehydrogenase. Biochim. Biophys. Acta 994, 187–190 (1989). 2910350 10.1016/0167-4838(89)90159-3

[b47] Vishwasrao H. D., Heikal A. A., Kasischke K. A. & Webb W. W. Conformational dependence of intracellular NADH on metabolic state revealed by associated fluorescence anisotropy. J. Biol. Chem. 280(26), 25119–25126 (2005). 15863500 10.1074/jbc.M502475200

[b48] Soldner F. *et al.* MPP^+^ inhibits proliferation of PC12 cells by a p21^WAF1/Cip1^-dependent pathway and induces cell death in cells lacking p21^WAF1/Cip1^. Exp. Cell Res. 250(1), 75–85 (1999). 10388522 10.1006/excr.1999.4504

[b49] Williams Z. R., Goodman C. B. & Soliman K. F. Anaerobic glycolysis protection against 1-methy-4-phenyl-pyridinium (MPP^+^) toxicity in C6 glioma cells. Neurochem. Res. 32, 1071–1080 (2007). 17401669 10.1007/s11064-006-9276-7

[b50] Schweitzer D. *et al.* J. Biomed. Opt. 9, 1214–1222 (2004). 15568942 10.1117/1.1806833

[b51] Huang S., Heikal A. A. & Webb W. W. Two-photon fluorescence spectroscopy and microscopy of NAD(P)H and flavoprotein. Biophys. J. 82, 2811–2825 (2002). 11964266 10.1016/S0006-3495(02)75621-X PMC1302068

[b52] Patterson G. H., Knobel S. M., Arkhammar P. & Thastrup O. & Piston, D. W. Separation of the glucose-stimulated cytoplasmic and mitochondrial NAD(P)H responses in pancreatic islet β cells. Proc. Natl. Acad. Sci. USA 97(10), 5203–5207 (2000). 10792038 10.1073/pnas.090098797 PMC25806

[b53] Li D., Zheng W. & Qu J. Y. Time-resolved spectroscopic imaging reveals the fundamentals of cellular NADH fluorescence. Opt. Lett. 33(20), 2365–2367 (2008). 18923624 10.1364/ol.33.002365

[b54] Conklin M. W., Provenzano P. P., Eliceiri K. W., Sullivan R. & Keely P. J. Fluorescence lifetime imaging of endogenous fluorophores in histopathology sections reveals differences between normal and tumor epithelium in carcinoma *in situ* of the breast. Cell Biocehm. Biophys. 53(3), 145–157 (2009). 10.1007/s12013-009-9046-7 PMC5575754 19259625

[b55] Xu C., Williams R. M., Zipfel W. & Webb W. W. Multiphoton excitation cross-sections of molecular fluorophores. Bioimaging 4, 198–207 (1996).

[b56] Levitt J. M., McLaughlin-Dublin M. E., Münger K. & Georgakoudi I. Automated biochemical, morphological, and organizational assessment of precancerous changes from endogenous two-photon fluorescence changes. Plos One 6(9), e24765 (2011). 21931846 10.1371/journal.pone.0024765 PMC3170385

[b57] Wang H.-W., Wei Y.-H. & Guo H.-W. Reduced nicotinamide adenine dinucleotide (NADH) fluorescence for the detection of cell death. Anti-cancer Agents Med. Chem. 9, 1012–1017 (2009). 10.2174/187152009789377718 19663784

[b58] Konig K. & Riemann I. High-resolution multiphoton tomography of human skin with subcellular spatial resolution and picosecond time resolution. J. Biomed. Opt. 8, 432–439 (2003). 12880349 10.1117/1.1577349

