# Supplementary material for: Quantification of the Metabolic State in Cell-Model of Parkinson’s Disease by Fluorescence Lifetime Imaging Microscopy
Source: Sci Rep. 2016 Jan 13;6:19145. doi: 10.1038/srep19145 (PMC4725947; doi:10.1038/srep19145)
Supplement: Supplementary Information [file srep19145-s1.pdf]

Supplementary Information

FULL ARTICLE

Quantification of the Metabolic State in Cell-Model of Parkinson's Disease by Fluorescence Lifetime Imaging Microscopy

Sandeep Chakraborty[1], Fang-Shin Nian[2,3], Jin-Wu Tsai[3,4], Artashes Karmenyan[**4,5], and Arthur Chiou[*1, 4]

[1]Institute of Biophotonics, National Yang-Ming University, Taipei, Taiwan 11221, ROC.

[2]Program in Molecular Medicine, National Yang-Ming University and Academia Sinica, Taipei, Taiwan 11529, ROC.

[3]Institue of Brain Science, National Yang-Ming University, Taipei, Taiwan 11221, ROC.

[4]Biophotonics and Molecular Imaging Research Center, National Yang-Ming University, Taipei, Taiwan 11221, ROC.

[5]Department of Physics, National Dong Hwa University, Hualien, Taiwan 97401, ROC.

*Corresponding author: e-mail: aechiou@ym.edu.tw , Tel: +886-2-2823-7141, Fax: +886-2-2823-6314.

**Corresponding author: e-mail: artashes@mail.ndhu.edu.tw, Tel: +886-3-863-3700, Fax: +886-3-863-0115.

Supplementary Figures

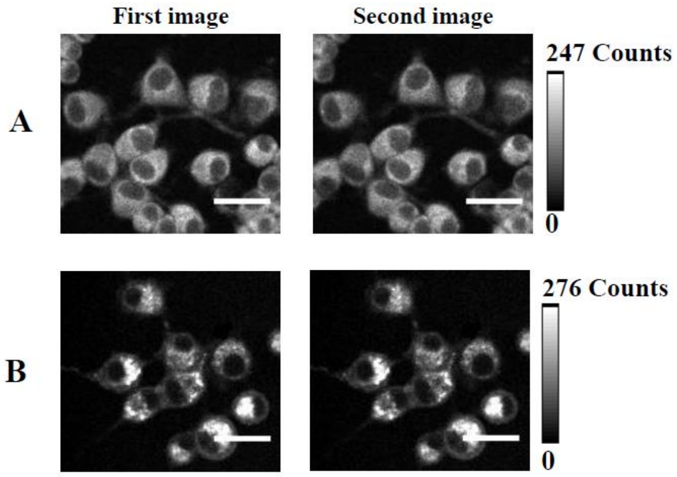

Figure S1. Photobleaching experiment. Upper panel A shows two successively taken intensity images of NADH at 760 nm excitation; likewise, lower panel B shows two consecutively taken intensity images of FAD at 860 nm excitation. In each case, the acquisition time for each image is 800 secs. The laser power was maintained around ~ 5 mW after the objective. The gray scales on the right represent the intensity. Scale bar: 20 µm. From these images, it can be seen that, in both cases, the intensity as well as the intensity distribution remains approximately the same for two successive images. This indicates that the image acquisition does not induce photobleaching effect. In this study, to further reduce the possible effect of photobleaching, each and every image was taken from different groups of cells for data analysis.

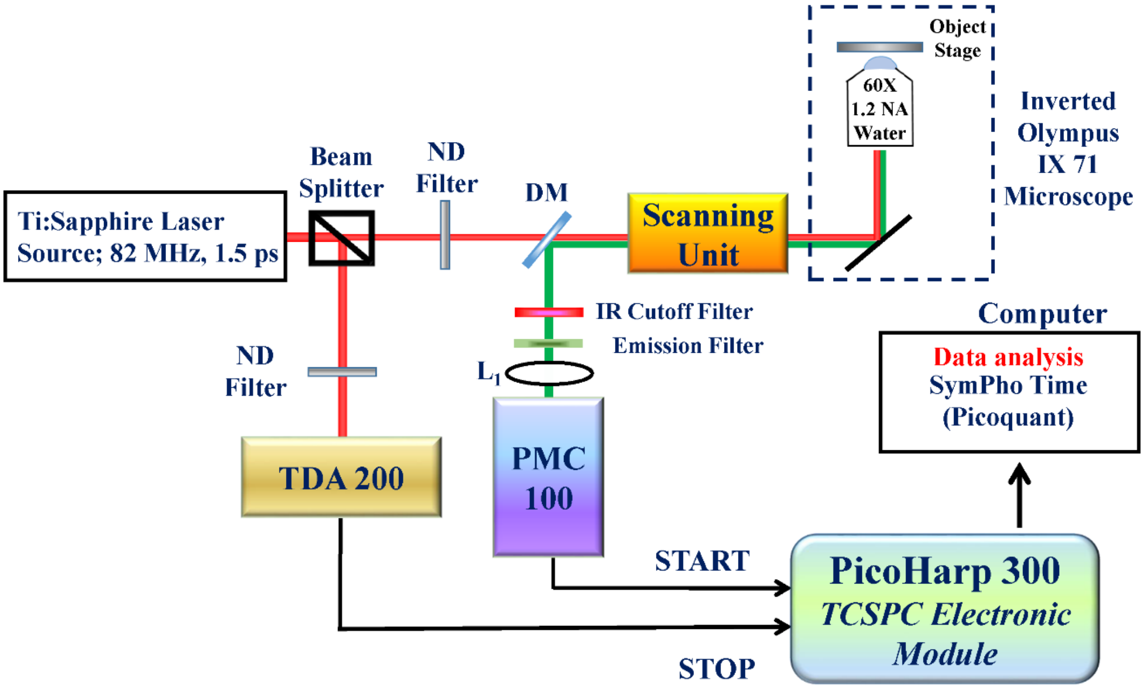

Figure S2: A schematic diagram of two-photon fluorescence lifetime imaging microscopy (2P-FLIM) set up. ND filter: Neutral density filter; DM: Dichroic mirror; L_1: focusing lens; TDA 200: photodiode; PMT: photomultiplier tube; PicoHarp 300: electronic board for time-correlated single photon counting (TCSPC). The details of the setup are described in “Materials and methods” section.

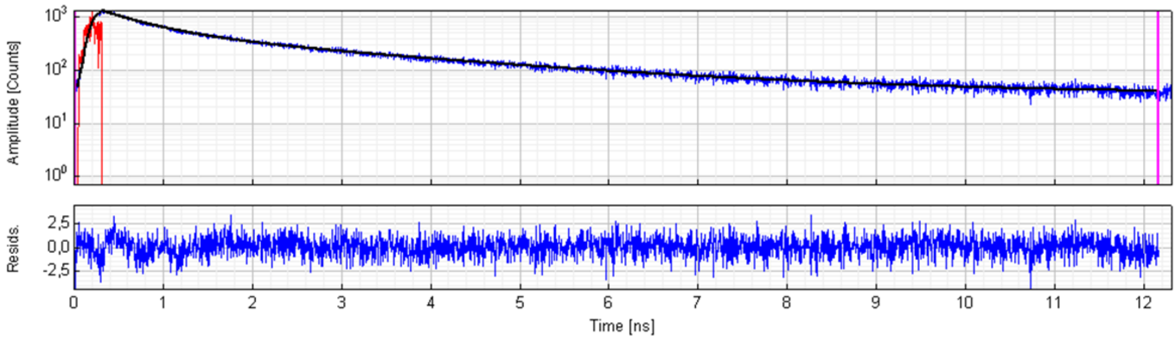

Figure S3. FLIM data analysis. Upper: Double-exponential fitting of NADH fluorescence lifetime decay curve (blue); the red curve is the instrument response function (IRF), while the black curve is the best fit. Lower: The residue does not exhibit any systematic variation; the chi square (χ^2) value remains approximately equal to 1. Details are given in “Materials and methods” section.

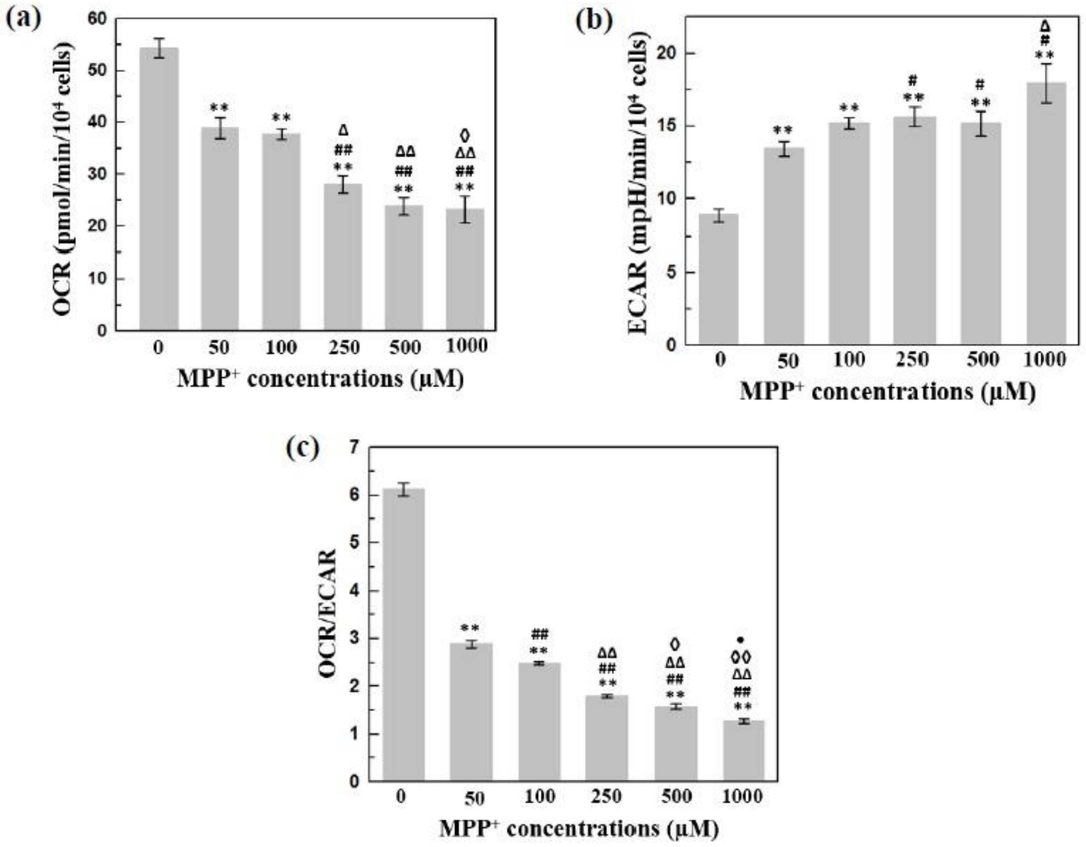

Figure S4: Cellular metabolic characterization of neuronal cells (derived from PC12 cells) without vs. with MPP$^+$ treatment. (a) Basal oxygen consumption rate (OCR) and (b) basal extracellular acidification rate (ECAR) in MPP$^+$ treated cells measured by using Seahorse Bioscience XF24 Extracellular Flux Analyzer (Massachusetts, USA) with the standard protocol. OCR is a direct measure of mitochondrial respiration while ECAR is the measure of lactic acid formation during glycolysis. It can be seen from (a) that the OCR decreased by 57.26% ($p < 0.001$), and from (b) that the ECAR increased by 50.44% ($p < 0.001$), from control to 1000 µM MPP$^+$ treatment. The ratio of OCR/ECAR also significantly ($p < 0.001$) decreased from control to 1000 µM MPP$^+$ treatment (c). These results strongly suggest the shifting of cellular ATP production from oxidative phosphorylation to anaerobic glycolysis. Each data point represents the mean ± SEM (n = 24) obtained from three independent experiments. One-way ANOVA with LSD post-hoc analysis was performed to statistically compare the different MPP$^+$ treated groups. Statistical significance: "**" : $p < 0.001$, for control (0 µM MPP$^+$) vs.

MPP^+ treated cells; "#" : $p < 0.05$, "##" : $p < 0.001$, for 50 μM MPP^+ treatment vs. other MPP^+ treated cells; "Δ" : $p < 0.05$, "ΔΔ" : $p < 0.001$, for 100 μM MPP^+ treatment vs. other MPP^+ treated cells; "◊" : $p < 0.05$, "◊◊" : $p < 0.001$, for 250 μM MPP^+ treatment vs. other MPP^+ treated cells; "●" : $p < 0.05$, for 500 vs. 1000 μM MPP^+ treatment.

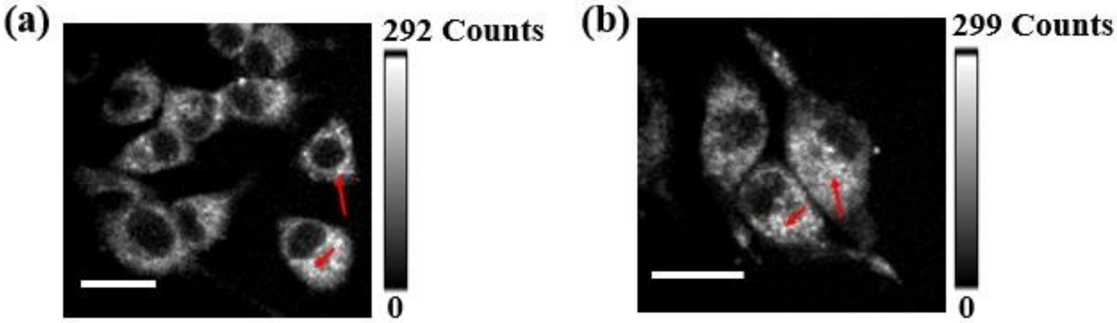

Figure S5. NADH fluorescence intensity images of PC12 cells at different magnifications. NADH intensity images of neuronal cells (derived from PC12 cells) using (a) 60x, water immersion, 1.2NA, and (b) 100x, oil immersion, 1.4NA objectives. Granular fluorescence intensity patterns in the cytoplasm are discernable, as indicated by red arrows. These granules are mitochondria as NADH autofluorescence from mitochondria dominates the fluorescence signal in this case. NADH fluorescence was excited with 760 nm via 2-photon excitation and the emission signal was collected with 447/60 nm band pass filter. The optical power at the object plane was ~ 5 mW. Scale bar: 20 μm.

Supplementary Tables

Table S1: Fluorescence lifetimes of free NADH (τ_1), protein-bound NADH (τ_2), and average of these weighted over their respective amplitudes (τ_{avg}) as well as ratio of the relative contributions of free- to protein-bound NADH components (a_1 / a_2) under different MPP^+ concentrations (0, 50, 100, 250, 500, and 1000 µM) treatment for five experimental days with average and SEM for each experimental day (15 data points) and the overall average and SEM of the all the imaging sessions of all the experimental days (75 data points).

Experimental Day (D)	Control				50 µM MPP^+ Treatment			
	τ_1 (ns) ± SEM	τ_2 (ns) ± SEM	τ_{avg}(ns) ± SEM	a_1/a_2 ± SEM	τ_1 (ns) ± SEM	τ_2 (ns) ± SEM	τ_{avg}(ns) ± SEM	a_1/a_2 ± SEM
**D = 1**	0.534 ± 0.012	2.615 ± 0.043	2.310 ± 0.030	0.842 ± 0.043	0.456 ± 0.005	2.361 ± 0.027	1.929 ± 0.033	1.308 ± 0.069
**D = 2**	0.510 ± 0.012	2.566 ± 0.043	2.197 ± 0.030	1.102 ± 0.028	0.451 ± 0.012	2.308 ± 0.049	1.96 ± 0.038	1.323 ± 0.054
**D = 3**	0.524 ± 0.012	2.553 ± 0.015	2.197 ± 0.018	0.977 ± 0.046	0.481 ± 0.010	2.403 ± 0.038	2.053 ± 0.043	1.187 ± 0.023
**D = 4**	0.510 ± 0.005	2.507 ± 0.025	2.168 ± 0.018	1.00 ± 0.028	0.473 ± 0.005	2.394 ± 0.023	2.034 ± 0.018	1.137 ± 0.038
**D = 5**	0.526 ± 0.005	2.566 ± 0.043	2.219 ± 0.020	1.103 ± 0.033	0.503 ± 0.010	2.503 ± 0.023	2.116 ± 0.018	1.235 ± 0.041
**Average of All the Imaging Sessions (75 data points)**	0.520 ± 0.005	2.566 ± 0.016	2.219 ± 0.013	1.002 ± 0.020	0.475 ± 0.004	2.403 ± 0.015	2.023 ± 0.015	1.230 ± 0.023

Experimental Day (D)	100 µM MPP^+ Treatment				250 µM MPP^+ Treatment			
	τ_1 (ns) ± SEM	τ_2 (ns) ± SEM	τ_{avg}(ns) ± SEM	a_1/a_2 ± SEM	τ_1 (ns) ± SEM	τ_2 (ns) ± SEM	τ_{avg}(ns) ± SEM	a_1/a_2 ± SEM
**D = 1**	0.448 ± 0.007	2.369 ± 0.025	1.907 ± 0.030	1.513 ± 0.080	0.443 ± 0.002	2.277 ± 0.028	1.952 ± 0.038	1.508 ± 0.087
**D = 2**	0.458 ± 0.005	2.327 ± 0.025	1.971 ± 0.028	1.191 ± 0.067	0.464 ± 0.007	2.358 ± 0.030	1.955 ± 0.018	1.303 ± 0.051
**D = 3**	0.487 ± 0.007	2.499 ± 0.025	2.050 ± 0.020	1.111 ± 0.033	0.489 ± 0.007	2.423 ± 0.025	2.107 ± 0.015	1.239 ± 0.041
**D = 4**	0.472 ± 0.005	2.308 ± 0.028	2.055 ± 0.030	1.159 ± 0.028	0.442 ± 0.005	2.407 ± 0.041	1.834 ± 0.030	1.824 ± 0.074
**D = 5**	0.466 ± 0.010	2.362 ± 0.015	2.039 ± 0.023	1.195 ± 0.033	0.452 ± 0.002	2.411 ± 0.025	1.996 ± 0.018	1.233 ± 0.111
**Average of All the Imaging Sessions (75 data points)**	0.467 ± 0.003	2.390 ± 0.012	2.006 ± 0.014	1.245 ± 0.032	0.462 ± 0.003	2.375 ± 0.015	2.003 ± 0.013	1.314 ± 0.031

Experimental Day (D)	500 µM MPP^+ Treatment				1000 µM MPP^+ Treatment			
	τ_1 (ns) ± SEM	τ_2 (ns) ± SEM	τ_{avg}(ns) ± SEM	a_1/a_2 ± SEM	τ_1 (ns) ± SEM	τ_2 (ns) ± SEM	τ_{avg}(ns) ± SEM	a_1/a_2 ± SEM
**D = 1**	0.458 ± 0.012	2.306 ± 0.041	1.914 ± 0.043	1.384 ± 0.095	0.423 ± 0.007	2.170 ± 0.028	1.756 ± 0.038	1.625 ± 0.095
**D = 2**	0.415 ± 0.005	2.232 ± 0.015	1.922 ± 0.012	1.107 ± 0.036	0.387 ± 0.002	2.056 ± 0.010	1.784 ± 0.007	1.302 ± 0.012
**D = 3**	0.464 ± 0.007	2.417 ± 0.028	2.011 ± 0.018	1.365 ± 0.036	0.433 ± 0.007	2.276 ± 0.038	1.858 ± 0.038	1.560 ± 0.069
**D = 4**	0.428 ± 0.002	2.240 ± 0.023	1.839 ± 0.018	1.489 ± 0.077	0.442 ± 0.005	2.308 ± 0.028	1.834 ± 0.030	1.824 ± 0.074
**D = 5**	0.457 ± 0.005	2.387 ± 0.025	2.037 ± 0.023	1.156 ± 0.041	0.445 ± 0.005	2.369 ± 0.025	1.896 ± 0.028	1.733 ± 0.087
**Average of All the Imaging Sessions (75 data points)**	0.446 ± 0.004	2.316 ± 0.016	1.937 ± 0.015	1.332 ± 0.035	0.431 ± 0.004	2.246 ± 0.018	1.823 ± 0.017	1.611 ± 0.046

Table S2: Fluorescence lifetimes of protein-bound FAD (τ_1), free FAD (τ_2), and average of these weighted over their respective amplitudes (τ_{avg}) as well as ratio of the relative contributions of free- to protein-bound NADH components (a_2 / a_1) under different MPP$^+$ concentrations (0, 50, 100, 250, 500, and 1000 µM) treatment for five experimental days with average and SEM for each experimental day (15 data points) and the overall average and SEM of the all the imaging sessions of all the experimental days (75 data points).

Experimental Day (D)	Control				50 µM MPP$^+$ Treatment			
	τ_1 (ns) ± SEM	τ_2 (ns) ± SEM	τ_{avg}(ns) ± SEM	a_2/a_1 ± SEM	τ_1 (ns) ± SEM	τ_2 (ns) ± SEM	τ_{avg}(ns) ± SEM	a_2/a_1 ± SEM
**D = 1**	0.464 ± 0.002	2.317 ± 0.069	2.012 ± 0.064	1.087 ± 0.111	0.346 ± 0.007	2.095 ± 0.020	1.846 ± 0.020	0.951 ± 0.051
**D = 2**	0.493 ± 0.020	2.372 ± 0.041	2.108 ± 0.038	1.283 ± 0.059	0.283 ± 0.007	2.092 ± 0.007	1.843 ± 0.010	0.945 ± 0.129
**D = 3**	0.338 ± 0.005	2.163 ± 0.030	1.840 ± 0.028	0.743 ± 0.030	0.374 ± 0.005	2.084 ± 0.018	1.728 ± 0.015	0.952 ± 0.103
**D = 4**	0.402 ± 0.015	2.223 ± 0.023	2.031 ± 0.018	1.633 ± 0.100	0.381 ± 0.005	2.097 ± 0.018	1.874 ± 0.020	0.941 ± 0.051
**D = 5**	0.433 ± 0.007	2.248 ± 0.028	1.853 ± 0.018	0.708 ± 0.025	0.389 ± 0.007	2.106 ± 0.002	1.798 ± 0.018	0.934 ± 0.025
**Average of All the Imaging Sessions (75 data points)**	0.435 ± 0.011	2.285 ± 0.023	2.001 ± 0.049	1.130 ± 0.105	0.327 ± 0.010	2.092 ± 0.015	1.819 ± 0.020	0.947 ± 0.085

Experimental Day (D)	100 µM MPP$^+$ Treatment				250 µM MPP$^+$ Treatment			
	τ_1 (ns) ± SEM	τ_2 (ns) ± SEM	τ_{avg}(ns) ± SEM	a_2/a_1 ± SEM	τ_1 (ns) ± SEM	τ_2 (ns) ± SEM	τ_{avg}(ns) ± SEM	a_2/a_1 ± SEM
**D = 1**	0.318 ± 0.012	2.024 ± 0.018	1.840 ± 0.018	0.957 ± 0.129	0.292 ± 0.005	2.038 ± 0.010	1.736 ± 0.010	1.001 ± 0.113
**D = 2**	0.316 ± 0.007	2.133 ± 0.023	1.667 ± 0.015	0.834 ± 0.028	0.361 ± 0.010	2.110 ± 0.030	1.850 ± 0.033	0.826 ± 0.033
**D = 3**	0.290 ± 0.002	2.025 ± 0.010	1.899 ± 0.002	0.551 ± 0.010	0.279 ± 0.002	2.005 ± 0.010	1.722 ± 0.020	0.584 ± 0.023
**D = 4**	0.334 ± 0.007	2.087 ± 0.010	1.873 ± 0.012	1.135 ± 0.038	0.321 ± 0.005	2.087 ± 0.018	1.771 ± 0.018	0.994 ± 0.038
**D = 5**	0.321 ± 0.005	2.107 ± 0.023	1.853 ± 0.005	0.962 ± 0.043	0.289 ± 0.010	2.057 ± 0.007	1.849 ± 0.007	0.972 ± 0.046
**Average of All the Imaging Sessions (75 data points)**	0.321 ± 0.010	2.070 ± 0.023	1.779 ± 0.028	0.894 ± 0.077	0.317 ± 0.007	2.057 ± 0.020	1.781 ± 0.025	0.876 ± 0.092

Experimental Day (D)	500 µM MPP^+ Treatment				1000 µM MPP^+ Treatment			
	τ_1 (ns) ± SEM	τ_2 (ns) ± SEM	τ_{avg} (ns) ± SEM	a_2/a_1 ± SEM	τ_1 (ns) ± SEM	τ_2 (ns) ± SEM	τ_{avg}(ns) ± SEM	a_2/a_1 ± SEM
**D = 1**	0.315 ± 0.007	2.105 ± 0.018	1.775 ± 0.020	0.939 ± 0.038	0.316 ± 0.012	2.070 ± 0.046	1.821 ± 0.043	0.743 ± 0.036
**D = 2**	0.309 ± 0.007	1.975 ± 0.012	1.835 ± 0.020	0.944 ± 0.082	0.295 ± 0.002	2.067 ± 0.028	1.802 ± 0.028	0.983 ± 0.098
**D = 3**	0.261 ± 0.009	2.093 ± 0.010	1.647 ± 0.010	0.518 ± 0.010	0.269 ± 0.002	2.068 ± 0.018	1.738 ± 0.018	0.673 ± 0.030
**D = 4**	0.289 ± 0.018	2.149 ± 0.005	1.850 ± 0.007	0.947 ± 0.005	0.264 ± 0.005	1.914 ± 0.010	1.721 ± 0.010	0.759 ± 0.012
**D = 5**	0.327 ± 0.005	2.110 ± 0.010	1.790 ± 0.010	0.915 ± 0.010	0.287 ±0.002	2.079 ± 0.007	1.814 ± 0.007	0.792 ± 0.005
**Average of All the Imaging Sessions (75 data points)**	0.301 ± 0.007	2.057 ± 0.020	1.783 ± 0.025	0.848 ± 0.067	0.293 ± 0.010	2.038 ± 0.036	1.775 ± 0.033	0.824 ± 0.072
